# Cloning and Comparative Studies of Seaweed Trehalose-6-Phosphate Synthase Genes

**DOI:** 10.3390/md8072065

**Published:** 2010-07-06

**Authors:** Guoliang Wang, Ge Zhao, Yanbin Feng, Jinsong Xuan, Jianwei Sun, Baotai Guo, Guoyong Jiang, Manli Weng, Jianting Yao, Bin Wang, Delin Duan, Tao Liu

**Affiliations:** 1 Institute of Genetics and Developmental Biology, Chinese Academy of Sciences, Beijing 100101, China; E-Mails: mgbl_01@ouc.edu.cn (G.W.); cathyge@163.com (G.Z.); fyb802@yahoo.com.cn (Y.F.); bnuxuan@hotmail.com (J.X.); jwsun@genetics.ac.cn (J.S.); mlweng@genetics.ac.cn (M.W.); bwang@genetics.ac.cn (B.W.); 2 College of Marine Life Science, Ocean University of China, Qingdao 266003, China; E-Mails: liutao@ouc.edu.cn (T.L.); mgbl_01@ouc.edu.cn (G.W.); 3 College of Life Sciences, Qingdao Agricultural University, Qingdao 266109, China, E-Mails: btguo@qau.edu.cn (B.G.); gyjiang@qau.edu.cn (G.J.); 4 Institute of Oceanology, Chinese Academy of Sciences, Qingdao 266071, China; E-Mail: yaojianting@ms.qdio.ac.cn (J.Y.)

**Keywords:** comparative genomics of TPS genes, gene cloning, RACE-PCR, seaweed, trehalose-6-phosphate synthase gene

## Abstract

The full-length cDNA sequence (3219 base pairs) of the trehalose-6-phosphate synthase gene of *Porphyra yezoensis* (*PyTPS*) was isolated by RACE-PCR and deposited in GenBank (NCBI) with the accession number AY729671. *PyTPS* encodes a protein of 908 amino acids before a stop codon, and has a calculated molecular mass of 101,591 Daltons. The PyTPS protein consists of a TPS domain in the N-terminus and a putative TPP domain at the C-terminus. Homology alignment for *PyTPS* and the TPS proteins from bacteria, yeast and higher plants indicated that the most closely related sequences to *PyTPS* were those from higher plants (OsTPS and AtTPS5), whereas the most distant sequence to PyTPS was from bacteria (EcOtsAB). Based on the identified sequence of the *PyTPS* gene, PCR primers were designed and used to amplify the *TPS* genes from nine other seaweed species. Sequences of the nine obtained *TPS* genes were deposited in GenBank (NCBI). All 10 *TPS* genes encoded peptides of 908 amino acids and the sequences were highly conserved both in nucleotide composition (>94%) and in amino acid composition (>96%). Unlike the *TPS* genes from some other plants, there was no intron in any of the 10 isolated seaweed *TPS* genes.

## Introduction

*Porphyra* is one of the most important seaweeds. It has a global distribution and important economic value. In addition to its roles in protecting aquatic ecosystems and as sources of food, biochemicals, pharmaceuticals [1,2] and bioenergy [3,4], *Porphyra* is now considered the best model organism for molecular biology research [5,6] and genomic research of seaweed [7]. However, molecular biological research in seaweeds is far behind the land plants and only a few nuclear genes have been described and cloned [2].

Trehalose (α-d-glucopyranosyl-(1,1)-α-d-glucopyranoside) is a non-reducing disaccharide of two glucose units presented throughout the animal, fungal, bacterial, yeast and plant kingdom [8,9], and functions as a stress protection metabolite in the stabilization of biological structures under stress tolerance and as a storage carbohydrate in plants [10,11]. The biosynthesis of trehalose has been studied in-depth in *Escherichia coli* (*E. coli*) and *Saccharomyces cerevisiae* (*S. cerevisiae*) and involves a two-step process catalyzed by trehalose-6-phosphate synthase (TPS) and trehalose-6-phosphate phosphatase (TPP). Trehalose-6-phosphate (T6P) has a critical role in plant growth and development; it is indispensable for carbohydrate utilization and growth in *Arabidopsis thaliana* (*A. thaliana*) [11,12]. T6P is also recognized as a regulator of sugar metabolism in plants [13–16]. Recently, it was proved that T6P functions as an inhibitor of SnRK1, a central integrator of stress and metabolic signals, to promote biosynthetic reactions in growing tissues [13]. Vandesteene *et al.* found that *Arabidopsis* encodes a single trehalose-6-P synthase (TPS) next to a family of catalytically inactive TPS-like proteins that might fulfill specific regulatory functions in actively growing tissues [15]. Different aspects of plant trehalose metabolism and function have been extensively reviewed [13–15].

*TPS* genes have been cloned from *E. coli* [17], *Metarhizium anisopliae* [18], *S. cerevisiae* [19,20], *A. thaliana* [21,22] and *Selaginella lepidophylla* [23], but not yet from seaweed. In *Arabidopsis*, disruption of the first step of trehalose synthesis, catalyzed by *AtTPS1*, has lethal consequences, demonstrating its essential physiological role [24].

We are interested in the seaweed *TPS* genes for the following reasons: first, the *TPS* gene encodes an enzyme involved in trehalose biosynthesis, which may become a model in functional gene research in seaweed; second, some experiments have shown that *TPS* genes from microorganisms can be expressed in transgenic plants, and increase the drought or salt tolerance of transgenic plants [25–30]. In most plants, trehalose is present in trace amounts and does not accumulate, but their genome sequences contain trehalose biosynthesis gene families [13,15]. Considering the high-salt living conditions of seaweed, its *TPS* gene may confer higher resistance to environmental stress than the corresponding genes from microorganisms, and may have potential usage in crop breeding by gene transformation. Here we report the characterization and molecular cloning of the *TPS* gene from *Porphyra yezoensis (PyTPS)* by RACE (Rapid Amplification of cDNA Ends)-PCR and the comparative analysis between the *PyTPS* gene and the *TPS* genes from some other seaweed species and other organisms.

## 2. Materials and Methods

### 2.1. Seaweed materials

The filaments of *Porphyra yezoensis* (*P. yezoensis*) and *Porphyra haitanensis* were cultured in axenic filtered seawater for 6 weeks at 16 °C and 25 °C, respectively, before the free filaments were collected for RNA and DNA preparations. The isolated gametophytes (male and female) of *Laminaria japonica* (*L. japonica*) and *Undaria pinnatifid* were propagated at 7 °C for 6 weeks before RNA and DNA extraction. PESI (Provasoli’s Enriched Seawater type I) solution was used as the medium for all cultures [31]. Samples of *Gracilaria lemaneiformis* and *Sargassum henslowianum* were cultivated in a cultivation tank and harvested for RNA and DNA preparations. The seaweed materials of *Monostroma angicava*, *Ulva pertusa*, *Chondrus ocellatus* and *Enteromorpha prolifera* were collected at the intertidal areas along the Qingdao coast, China. After identification, the samples were washed and brushed several times with autoclaved seawater to eliminate the algal epiphytes. Finally, the clean seaweed materials were used for RNA and DNA extraction. The *P. yezoensis* cell line Qingdao-8 was used for *PyTPS* gene cloning.

### 2.2. Generation of the TPS gene from P. yezoensis

For RACE-PCR amplification, total RNA was extracted from filaments of cell line Qingdao-8 by a modified guanidine thiocyanate (GT) method [32]. In order to obtain a full-length cDNA sequence of the *PyTPS* gene, the SMART^TM^ RACE cDNA Amplification Kit (Clontech) was used according to the supplier’s protocol. Since a 453 base pair (bp) fragment of the *PyTPS* gene was already identified in our previous work [33], the gene-specific primers (GSP1 and GSP2) were designed for RACE reactions according to this sequence Primer GSP1 (5′-CTGTTCGCCTCGTGCTCCAGGTTAAG-3′) was used for generation of the 5′ end of *PyTPS*, while GSP2 (5′-GCATTGCCCTCAAGCTGATGGGTTTC-3′) and the following designed nested PCR primers NGSP2 (5′-GGTCGTACTTGTGCAAGTTGCCATCC-3′) and 2NGSP2 (5′-GACCTGTCATGGATGGAGTTGGCATTGC-3′) were used for generation of the 3′ end of *PyTPS*. The RACE-PCR products were cloned into the pMD-18T vector (TaKaRa, Dalian, China) for sequencing.

### 2.3. DNA extraction

Seaweed material was ground into powder in liquid nitrogen and then DNA was extracted with a plant genomic DNA extraction kit (Tianwei Biotech, Beijing, China) as in our previous report [34].

### 2.4. Total RNA extraction and cDNA syntheses

To prepare cDNA template for PCR amplifications, total RNA was extracted according to a modified GT method [35]. The RNA was quantified and checked at wavelengths of 260 nm and 280 nm and by formaldehyde RNA gel electrophoresis. Five μg of total RNA was then digested with DNase I (TaKaRa, Japan) followed by first-strand cDNA synthesis using the M-MLV reverse transcriptase (Promega, USA). The first-strand cDNA was used as a template in PCR amplifications.

### 2.5. PCR amplification of TPS genes from other nine seaweed species

The open reading frame (ORF) sequence of the seaweed *TPS* gene is about 2.7 kilo bases (kb). Based on the obtained cDNA sequence of *PyTPS*, three primer-pairs (Table 1) were designed and used to amplify the *TPS* gene from cDNA and genomic DNA of the other nine seaweeds. Their amplified fragments were about 1.3, 1.1 and 0.8 kb, respectively, and overlapped. Related primer information is provided in Table 1. PCR was conducted using the LA Taq^®^ system (TaKaRa); PCR products were confirmed by sequencing. After sequencing and assembly, the entire ORF sequences of *TPS* genes were identified.

### 2.6. Analyses and comparison of TPS genes

Analysis of the cDNA sequences was performed using the BLASTX search program (Version 2.2.21+) served by NCBI (http://www.ncbi.nlm.nih.gov/BLAST/). Multiple sequence alignments and cluster analysis of *TPS* genes were carried out by DNAMAN software (Version 6, Lynnon Corporation).

### 2.7. Cloning the PyTPS gene into vector pET22b

First, total RNA was used as a template to synthesize first-strand cDNA. The entire PyTPS gene was then generated from cDNA by RT-PCR using primers TPS-R1 (a 5′ primer incorporating an *Nde*1 site overlapping the *PyTPS* initiator ATG codon) and TPS-b2 (a 3′ primer with a *Hin*dIII site incorporated downstream of the *TPS* translation stop codon) (Table 1). PCR was conducted using the LA Taq^®^ system (TaKaRa) to generate a *~*2.7 kb fragment (*Nde*1-*Hin*dIII). After the amplified fragment was gel purified and digested with restriction enzymes *Nde*1 and *Hin*dIII (New England BioLabs, Inc.), the fragment was ligated into the pET22b vector (Novagen) using the *Nde*1 and *Hin*dIII sites to yield plasmid pET22b/*PyTPS.*

The plasmid pET22b/*PyTPS* was transformed into *E. coli* strain BL21(DE3) [36] for *PyTPS* overexpression. The transformants were grown in LB medium with ampicillin (50 μg/mL) at 37 °C to mid-logarithmic phase. *PyTPS* expression was induced by addition of 1mM IPTG (isopropylthio-β-d-galactoside) and growth was continued for 4 h at 37°C. An aliquot of 1 mL cells was harvested and resuspended in 150 μL TE and separated by SDS-polyacrylamide gel electrophoresis (PAGE; 7.5%).

## 3. Results

### 3.1. Generation of full-length PyTPS Cdna

Three successive rounds of RACE-PCR were performed to reach the 3′ end of the *PyTPS* gene, while only one round of RACE-PCR was performed to reach the 5′ end of the *PyTPS* gene. The RACE-PCR products were sequenced, analyzed and assembled by BLAST.

**Figure 1:**
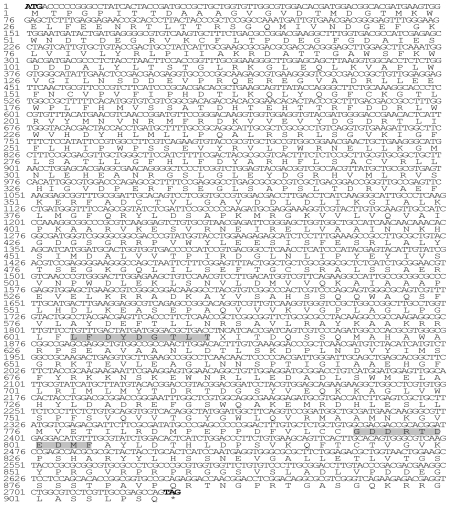
Nucleotide and deduced amino acid sequences of the *PyTPS* gene. The start and stop codons are shown in boldface. The amino acid residues 603–611 and 795–804 are the two typical conserved regions in the phosphatase family and are shaded here with a gray background.

After assembly, a 3219 bp full-length cDNA of the *PyTPS* gene was obtained, and then it was deposited in GenBank (NCBI) with the accession numbers AY729671 (mRNA) and AAW27916 (protein). AY729671 contains a 216 bp 5′-leader sequence upstream of the ATG initiation codon, 276 bp of 3′ UTR (untranslated region) downstream of the termination codon (TAG), and an ORF (2727 bp) coding the TPS protein of 908 amino acids and a stop codon with a calculated molecular mass of 101,591 Daltons. The nucleotide sequence of the coding region and the deduced amino acid sequence of the *PyTPS* gene are shown in Figure 1.

### 3.2. Expression of the PyTPS gene in E. coli

**Figure 2:**
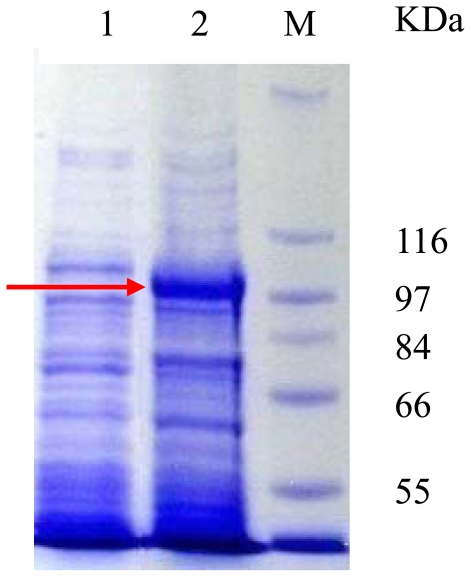
Over-expression of the *PyTPS* gene in *E. coli* strain BL21(DE3). 15 μL of cell lysate was fractionated in a SDS/7.5% polyacrylamide gel. Lane 1, cell lysate of *E. coli* transformed by pET22b; Lane 2, cell lysate of *E. coli* transformed by pET22b/*PyTPS*; M, molecular mass markers (Sigma). The gel was stained with Coomassie Brilliant Blue. Arrow points to the overexpressed PyTPS protein at ~101 kDa.

The plasmid pET22b/*PyTPS* was constructed and transformed into *E. coli* strain BL21(DE3). Electrophoresis results showed that a strong PyTPS protein band was observed in the sample carrying pET22b/*PyTPS* (Figure 2, lane 2), but that no band was found in the sample carrying pET22b (Figure 2, lane 1). The result proved that the *PyTPS* gene was highly expressed in the *E. coli* strain.

### 3.3. Domain analysis of the PyTPS protein

**Figure 3:**
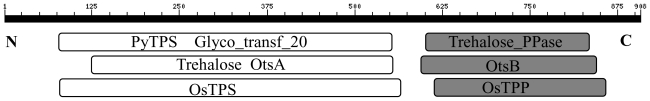
Block diagram of the TPS proteins from *P. yezoensis*, rice (*O. sativa*) and bacteria *(E. coli)*. The N-terminal TPS domain is marked as the light-colored boxes (left side), and the C-terminal TPP domain is marked as the dark-colored boxes (right). From top to bottom: the corresponding domains of the TPS proteins from *P. yezoensis, E. coli* and *O. sativa*.

The BLAST results showed that, similar to the TPS proteins from other higher origins, the deduced PyTPS protein consists of a TPS domain at the N-terminus and a putative TPP domain at the C-terminus (Figure 3). The PyTPP domain has two typical sequences (LFDYDGTLT and GDDRTDEDMF) at amino acid positions 603–611 and 795–804 of the TPS protein that are conserved regions in the phosphatase family [37,38].

### 3.4. Comparison of PyTPS with TPS proteins from other organisms

**Figure 4:**
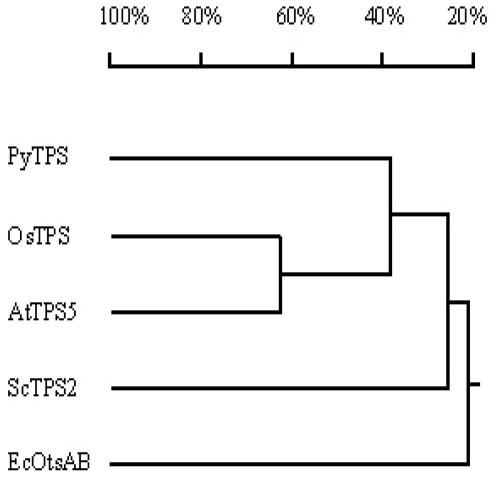
Dendrogram analysis of TPS proteins from five different organisms. The PyTPS (AAW27916) is compared with OsTPS (deduced from AAT01318.1), AtTPS5 (deduced from BAC43297.1), ScTPS2 (deduced from CAA50025.1) and EcOtsAB (deduced from NP_288332.1 and NP_288333.1).

The PyTPS protein deduced from the *PyTPS* gene (AY729671, *P. yezoensis*) and four other TPS proteins deduced from corresponding *TPS* genes of bacteria (*EcotsA* and *EcotsB*, NP_288332.1 and NP_288333.1, *E. coli*), yeast (*ScTPS2*, CAA50025.1, *S. cerevisiae*) and two model plants (*OsTPS*, AAT01318.1, *Oriza sativa*, and *AtTPS5*, BAC43297.1, *A. thaliana*) were compared. Results were plotted in a dendrogram (Figure 4). Alignment of these TPS proteins shows that PyTPS and AtTPS5 have the highest similarity with 37.7% identity; PyTPS and OsTPS have 37% identity; PyTPS and ScTPS2 have 27% identity; and PyTPS and EcOtsAB have only 20% identity.

### 3.5. Comparison of PyTPS with TPS genes from other nine seaweeds

In addition to *PyTPS*, the *TPS* genes were PCR amplified from nine other seaweed species. Three of them (*Porphyra haitanensis*, *Gracilaria lemaneiformis* and *Chondrus ocellatus*) are Rhodophyta; three (*Monostroma angicava*, *Ulva prolifera* and *Enteromorpha prolifera*) are Chlorophyta, and three (*Laminaria japonica*, *Undaria pinnatifida* and *Sargassum henslowianum*) are Phaeophyta. The nine *TPS* genes were successfully PCR amplified from cDNA and genomic DNA. The PCR products were sequenced and the identified *TPS* genes were deposited in GenBank (NCBI); their accession numbers are listed in Table 2.

**Figure 5:**
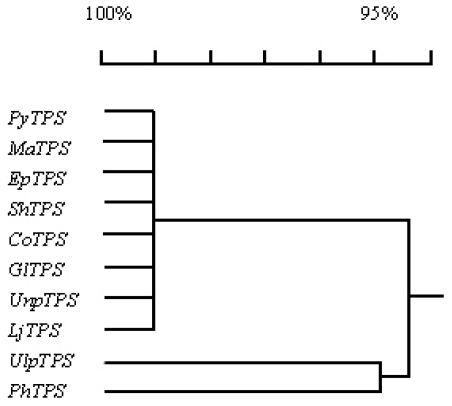
Dendrogram analysis of 10 seaweed *TPS* genes using the DNAMAN program. See Table 2 for nomenclature of the *TPS* genes in detail.

Comparison of nucleotide sequences of the *TPS* genes from the nine seaweed species with *PyTPS* indicated that all of these *TPS* genes contained an ORF with the same size of 2727 nucleotides. The identity of the 10 nucleotide sequences is higher than 94% (Table 3 and Figure 5).

### 3.6. Comparison of the TPS gene sequences from cDNA and genomic DNA

Nucleotide sequence comparison between cDNA and genomic DNA of the *TPS* genes from 10 different seaweed species indicated that the sequences from cDNA and from genomic DNA were identical, confirming that no intron existed in all of the 10 *TPS* genes investigated.

### 3.7. Comparison of the TPS proteins from the 10 seaweed species

**Figure 6:**
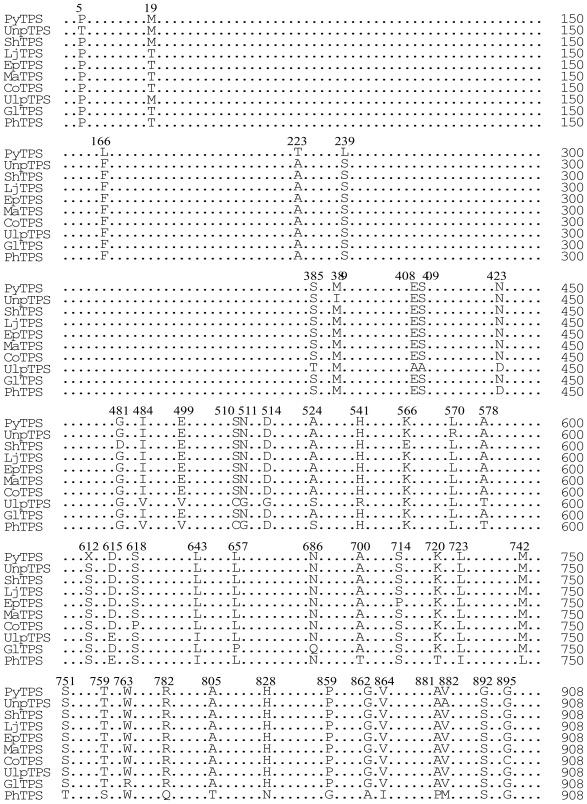
Multiple alignment of the amino acid sequence of the TPS proteins from 10 seaweed species. The dots indicate positions where identical amino acids were observed between the 10 seaweed TPS proteins. Varied amino acid residues are shown with their location numbers.

**Figure 7:**
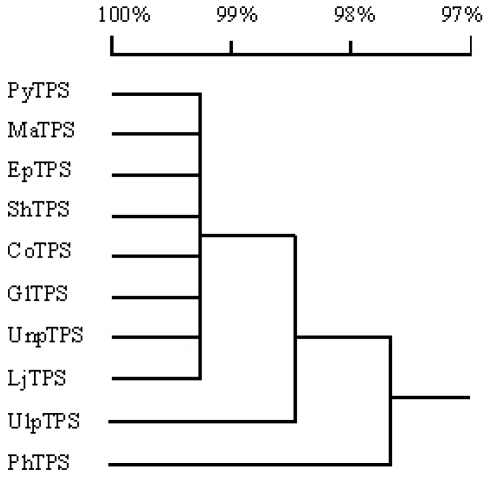
Polygenetic analysis of the 10 seaweed TPS proteins.

Comparison of the amino acid sequences of TPS proteins from 10 different seaweed species indicated that they were identical in size (908 amino acids); and that their sequences had an identity higher than 96% (Table 3, Figures 6 and 7).

## 4. Discussion

Trehalose might interfere with the sugar sensing mechanisms and other signal transduction pathways [39,40]. In *Selaginella lepidophylla*, trehalose forms glasses (vitrification) in the dry state for the stabilization of macromolecules [23]. The trehalose pathway is now known to be ubiquitous in plants [41]. The reported results proved that in *Arabidopsis* it is indispensable for carbohydrate utilization during plant growth and development [11,16].

Most plant *TPS* genes have introns. In the *A. thaliana* genome there are 11 *TPS* homologs: *AtTPS1~4* contain 16 introns and *AtTPS5~11* contain two or three introns [24,41]. In cultivated cotton (*Gossypium hirsutum L.*) the *TPS* gene was separated by two introns [38]. Sequence analysis indicated that, unlike the situation in *TPS* genes of higher plants, which have introns and exons in their genomic DNA sequences, there is no intron in any of the 10 seaweed *TPS* genes investigated in this study, which included species from red algae, brown algae and green algae. The *E. coli otsA* gene and yeast *TPS* genes are also without introns. This may reflect that seaweed belongs to lower plants in evolutionary taxonomy, and is very close to the prokaryote *E. coli* and the lower eukaryote yeast. Furthermore, the 10 *TPS* genes show highly conserved DNA sequences; their nucleotide sequence identity is higher than 94% (Figure 5); however the identity between seaweed and other organisms is much lower (Figure 4).

It has been reported that two plant *TPS* genes, *AtTPS1* and *SlTPS* cloned from *A. thaliana* and *Selaginella lepidophylla*, could partially complement an *S. cerevisiae tps1 Δ* mutant, but most plant *TPS* genes failed to complement the *S. cerevisiae tps1 Δ* mutant [21,23,42]. In addition, *AtTPPA* and *AtTPPB* were able to complement the yeast *tps2* mutant [22]. In our experiments the cloned *PyTPS* gene failed to complement the *tps1 Δ* and *tps2* mutant (data not shown). We think the reason may be due to the structure of *PyTPS* gene itself. By applying BLAST analysis to compare the protein sequences of PyTPS and the 11 TPS proteins from *Arabidopsis*, the results indicated that the highest identity was found between PyTPS and AtTPS7 (identity = 37.7%) and the lowest identity was found between PyTPS and AtTPS1 (identity = 27.7%). Vogel *et al.* [40] had reported that the AtTPS7 and AtTPS8, although expressed, appeared to lack both TPS and TPP activity in yeast transformants. We think the similarity between PyTPS and AtTPS7 may make PyTPS more like AtTPS7 in lacking TPS and TPP activity in yeast transformants.

In recent years, trehalose metabolism has been implicated with stress tolerance and the control of yeast glycolysis [41]. Some experiments have indicated that transgenic plants expressing *TPS* genes from microorganisms exhibited increased stress tolerance. Seaweed is a kind of lower plant and belongs to algae, which can synthesis and accumulate trehalose [15]. So far no report has characterized the seaweed *TPS* gene. In this study, firstly we cloned *TPS* gene from the seaweed *P. yezoensis*, and it was studied in comparison with subsequently isolated *TPS* genes from other nine seaweed species. The results reported here will be helpful for the continued study of the function of seaweed *TPS* gene in stress tolerance, and for exploring its possible application in stress tolerance breeding of grain plants by gene transformation.

Recently, the *PyTPS* gene has been transformed into cultivated rice by agro-bacterium mediated transformation in our laboratory and some transgenic lines show increased salt/drought tolerance [43]. This will have potential applications in crop breeding in the future.

## Figures and Tables

**Table 1:** Primers used for the isolation of seaweed *TPS* genes by PCR amplification.

Primer in pair	Sequence (5′→3′)	Product size (kb)	Orientation and position
1.TPSR1	GACTCATATG^a^ACCCCCGGGCCTATCACTA	1.36	5′→3′ (1~22 nt)
3KpnI	CATGATGCTGTACAGCGCAAG		3′→5′ (1339 ~1359 nt)
2.Tre1	CTACGCGCGTCACTTTCTCTC	1.2	5′→3′ (861~881 nt)
TPSa2	CACTCCTTCGAATTCTTCTTG		3′→5′ (2034~2054 nt)
3.TPSb1	CAAGAAGAATTCGAAGGAGTG	0.7	5′→3′ (2034~2054 nt)
TPSb2	GACTAAGCTT^b^CTACTGGCTCGGCAACGAGGAC		3′→5′ (2706~2727 nt)

a The *Nde*1 restriction site (underlined);

b The *Hin*dIII restriction site(underlined).

**Table 2:** Isolated seaweed *TPS* genes and their accession numbers in GenBank.

Isolated *TPS* gene	From the seaweed species	GenBank accession number (NCBI)
*PyTPS*	*Porphyra yezoensis*	AY729671
*MaTPS*	*Monostroma angicava*	DQ666324
*LjTPS*	*Laminaria japonica*	DQ666325
*PhTPS*	*Porphyra haitanensis*	DQ666326
*GlTPS*	*Gracilariopsis lemaneiformis*	DQ666327
*CoTPS*	*Chondrus ocellatus*	DQ666328
*UlpTPS*	*Ulva pertusa*	DQ666329
*EpTPS*	*Enteromorpha prolifera*	DQ666330
*UnpTPS*	*Undaria pinnatifida*	GQ352535
*ShTPS*	*Sargassum henslowianum*	GQ352536

**Table 3:** Variation of nucleotide and deduced amino acid sequences between the 10 seaweed *TPS* genes. (See Table 2 for the nomenclature of the *TPS* genes). The homologies of nucleotide sequences and their deduced amino acid sequences were calculated by comparing the differences in sequences between the indicated *TPS* and *PyTPS* or TPS and PyTPS, respectively.

*TPS* gene	Numbers of nucleotide variations	Numbers of amino acid substitutions	Homology of nucleotide sequences (%)	Homology of deduced amino acid sequences (%)
*PyTPS*	-	-	100	100
*MaTPS*	8	6	99.7	99.3
*EpTPS*	9	7	99.7	99.3
*ShTPS*	10	7	99.6	99.2
*CoTPS*	11	8	99.6	99.2
*GlTPS*	11	8	99.6	99.1
*UnpTPS*	14	9	99.5	99.1
*LjTPS*	28	6	99.0	99.0
*UlpTPS*	95	19	96.5	97.9
*PhTPS*	152	30	94.4	96.7
